# Gram-Scale Preparation of Cannflavin A from Hemp (*Cannabis sativa* L.) and Its Inhibitory Effect on Tryptophan Catabolism Enzyme Kynurenine-3-Monooxygenase

**DOI:** 10.3390/biology11101416

**Published:** 2022-09-28

**Authors:** Tess Puopolo, Tanran Chang, Chang Liu, Huifang Li, Xu Liu, Xian Wu, Hang Ma, Navindra P. Seeram

**Affiliations:** 1 Bioactive Botanical Research Laboratory, Department of Biomedical and Pharmaceutical Sciences, College of Pharmacy, University of Rhode Island, Kingston, RI 02881, USA; 2 George and Anne Ryan Institute for Neuroscience, University of Rhode Island, Kingston, RI 02881, USA; 3 State Key Laboratory of Biochemical Engineering, Institute of Process Engineering, Chinese Academy of Sciences, Beijing 100190, China; 4 University of Chinese Academy of Sciences, Beijing 100049, China; 5 Yunnan Hempmon Pharmaceutical Co., Ltd., Kunming 650032, China; 6 Department of Kinesiology, Nutrition, and Health, Miami University, Oxford, OH 45056, USA

**Keywords:** *Cannabis sativa* L., cannflavin A, kynurenine pathway, tryptophan, inflammation, neurodegeneration, binding affinity, surface plasmon resonance

## Abstract

**Simple Summary:**

Kynurenine-3-monooxygenase (KMO) is an enzyme in the neurotoxic branch of the kynurenine pathway (KP) and is a target of inhibitors with therapeutic potential against neuroinflammatory and neurodegenerative diseases. Phytochemicals from hemp (*Cannabis sativa* L.) including phytocannabinoids and flavonoids, can modulate enzymes involved in the KP metabolism, but their direct inhibition on KMO is unknown. In the current study, we purified a unique *C. sativa* flavonoid, namely, cannflavin A (CFA), from the hemp aerial material at a gram-scale. We evaluated the anti-KMO activity of CFA and a panel of phytocannabinoids. CFA was the most active compound with an IC_50_ of 29.4 μM, which was comparable to the positive control Ro 61-8048 (IC_50_ = 5.1 μM). We used a modeling method to demonstrate the interactions between CFA and the KMO protein and measured the binding affinity by a biophysical tool (surface plasmon resonance; SPR). We also conducted a competitive SPR assay to show that CFA and Ro 61-8048 may bind to the same location of the KMO protein. Our findings help the understanding of *C. sativa* compounds’ effects on KMO.

**Abstract:**

Inhibitors targeting kynurenine-3-monooxygenase (KMO), an enzyme in the neurotoxic kynurenine pathway (KP), are potential therapeutics for KP metabolites-mediated neuroinflammatory and neurodegenerative disorders. Although phytochemicals from *Cannabis* (*C. sativa* L.) have been reported to show modulating effects on enzymes involved in the KP metabolism, the inhibitory effects of *C. sativa* compounds, including phytocannabinoids and non-phytocannabinoids (i.e., cannflavin A; CFA), on KMO remain unknown. Herein, CFA (purified from hemp aerial material at a gram-scale) and a series of phytocannabinoids were evaluated for their anti-KMO activity. CFA showed the most active inhibitory effect on KMO, which was comparable to the positive control Ro 61-8048 (IC_50_ = 29.4 vs. 5.1 μM, respectively). Furthermore, a molecular docking study depicted the molecular interactions between CFA and the KMO protein and a biophysical binding assay with surface plasmon resonance (SPR) technique revealed that CFA bound to the protein with a binding affinity of $4.1\times 10^{-5}\;M$. A competitive SPR binding analysis suggested that CFA and Ro 61-8048 bind to the KMO protein in a competitive manner. Our findings show that *C. sativa* derived phytochemicals, including CFA, are potential KMO inhibitors, which provides insight into the development of therapeutics targeting the KP and its related pathological conditions.

## 1. Introduction

**Figure 1:**
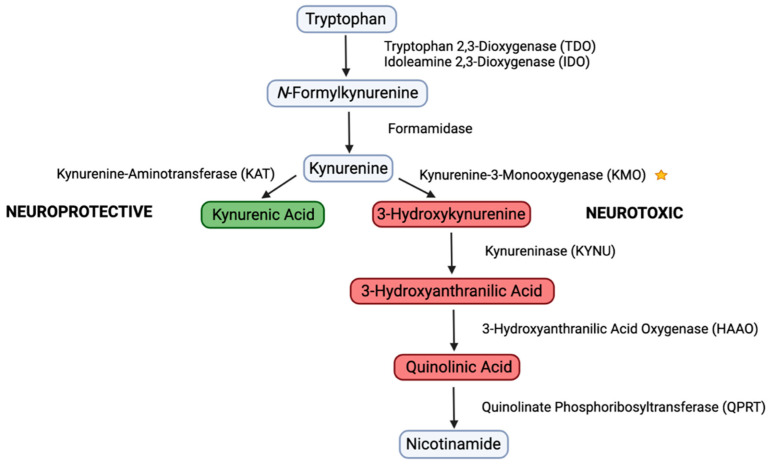
Schematic representation of the kynurenine pathway (KP) consisting of neuroprotective and neurotoxic branches. The downstream neurotoxic products are a target for inhibitors which aim to shift the pathway towards neuroprotective products. The star symbol indicates the enzyme under the current study.

Tryptophan, an essential amino acid, has been well characterized for its role in the biosynthesis of melatonin and serotonin. However, the catabolism of tryptophan into the oxidized nicotinamide adenine dinucleotide (NAD^+^) by the kynurenine pathway (KP) in a normal physiologic state is less studied [1]. The KP consists of two branches, which lead to the generation of either neuroprotective or neurotoxic products [2,3] (Figure 1). First, in the rate limiting step, tryptophan is oxidized into N-formylkynurenine by tryptophan 2,3-dioxygenase (TDO) or idoleamine 2,3-dioxygenase (IDO) [3]. Deformylated N-formylkynurenine is further deformylated by formamididase to produce kynurenine [3]. Kynurenine serves as the branching point and is subsequently converted into opposing fates, either neuroprotective or neurotoxic [3]. The neuroprotective branch produces kynurenic acid, which acts as a non-competitive antagonist of α7 nicotinic acetylcholine (α7nACh) receptors and exhibits antioxidant activities [4,5]. The neurotoxic branch involves the conversion of kynurenine to 3-hydroxykynurenine by kynurenine-3-monooxygase (KMO), 3-hydroxyanthranilic acid by 3-hydroxyanthranilic acid oxygenase (HAAO), and quinolic acid by quinolinate phosphoribosyltransferase (QPRT), leading to the production of NAD^+^ [4]. Dysregulation of the KP neurotoxic metabolites have been implicated in the progression of disorders, including neurodegenerative diseases, inflammation, cardiovascular disease, chronic pain, depression, and cancer [4,6,7,8].

Due to the accumulation of neurotoxic products in numerous detrimental conditions, the enzymes involved in the neurotoxic branch of the KP are a target for the development of therapeutic interventions [9]. KMO is a flavin dependent hydroxylase fundamental to the neurotoxic branch of the KP [1]. KMO utilizes NADPH and a flavin adenine dinucleotide (FAD) cofactor to convert kynurenine to 3-hydroxykynurenine which acts to produce additional neurotoxic metabolites [1]. KMO is localized to the outer mitochondrial membrane and has been identified in peripheral tissues, macrophages, and monocytes, as well as microglial cells, neurons and astrocytes in the central nervous system [1].

Inhibitors of KMO are of therapeutic interest due to their anticipated shift in the KP from neurotoxic to neuroprotective metabolites by limiting the production of 3-hydroxykynurenine, 3-hydroxyanthranillic acid, and quinolinic acid [1]. Additionally, KMO inhibitors may modulate the disease state by increasing the production of the neuroprotective product kynurenic acid [1,10]. A number of KMO inhibitors have been discovered, both similar and dissimilar to the enzyme substrate L-Kyn. *m*-nitrobenzoyl alanine was the first specific KMO inhibitor discovered (IC_50_ = 0.9 μM) with effects of reducing 3-hydroxykynurenine and increasing kynurenic acid [1,11]. A potent KMO inhibitor, UPF-648 IC_50_ = 40 nM) showed similar effects in increasing the neuroprotective products over neurotoxic [11]. A well characterized inhibitor, Ro 61-8048, was identified to have an IC_50_ of 37 nM in rat liver and kidney homogenates in vitro [11]. Ro 61-8048 also demonstrated neuroprotective effects in a model of levodopa-induced parkinsonism in monkeys via KMO inhibition [11]. Further, it was reported that Ro 61-8048 exerted neuroprotection from the periphery as it cannot cross the blood brain barrier [12].

Despite preclinical studies involving the discovery of KMO inhibitors, KMO inhibitors have failed to reach clinical development as of 2017 [11]. Therefore, further evaluation of compounds with inhibitory effects on KMO are warranted. Among these compounds, natural products are promising potential inhibitors of the KP enzymes. For instance, an extract of a sponge of the *Aka* species and its isolated compound, serotonin sulfate, were reported to inhibit the enzyme kynureninase [13]. Additionally, a prenylated flavonoid monosaccharide, namely, icariside I, from *Epimedium* spp. was shown to block the kynurenine pathway by downregulating KMO mRNA, demonstrating implications for cancer immunotherapy [14]. Lastly, several naturally derived compounds, including curcumin, resveratrol, and quercetin have exhibited inhibitory effects on IDO [15]. Yet, whether naturally derived compounds can inhibit KMO activity remains understudied.

*C. sativa* contains a group of unique bioactive phytochemicals, namely, phytocannabinoids, including the major ones, such as hallucinogenic tetrahydrocannabinol (THC) and non-hallucinogenic cannabidiol (CBD), as well as a group of minor phytocannabinoids, e.g., cannabitriol (CBT), cannabigerol (CBG), cannabidivarin (CBDV), cannabinol (CBN), cannabichromene (CBC), and cannabigerolic acid (CBGA). Published studies support that these compounds may exert anti-inflammatory and neuroprotective effects. For instance, our group has previously reported CBD’s anti-inflammatory action associated with P2X7, the inhibitory effects of CBD on alpha-glucosidase, and the inhibitory action of CBD and a panel of minor cannabinoids on acetylcholinesterase and butyrylcholinesterase [16,17,18]. Apart from the phytocannabinoid content, *C. sativa* also contains numerous non-phytocannabinoid types of phytochemicals, such as the flavonoid cannflavin A (CFA). Although the levels of CFA in different *C. sativa* extracts are low, CFA has shown various biological activities [19]. CFA has been found to exert promising anti-inflammatory effects by inhibiting pro-inflammatory enzymes, including prostaglandin E2 and cytochrome c oxidases I and II, as well as shown neuroprotective properties in PC12 cells [20]. However, the effect of CFA against the neurotoxic arm of the KP, specifically KMO, remains unknown, which is at least partially due to the barrier of its feasibility given its low natural abundance in *C. sativa.* Herein, we aimed to develop a solvent extraction method with a combination of chromatographic techniques to purify CFA from hemp material at a gram-scale. In addition, CFA and CBD, along with a panel of minor phytocannabinoids, were screened for the potential inhibitory effects on KMO activity using an enzymatic assay. The mechanism of action (i.e., the target binding mode) of the identified KMO inhibitor was studied with an *in-silico* approach (molecular docking) and a label-free biosensor-based binding assay (surface plasmon resonance; SPR).

## 2. Materials and Methods

### 2.1. Chemicals and Reagents

A screening assay kit for KMO inhibitors was obtained from BPS Bioscience (San Diego, CA, USA). Ro 61-8048 (purity ≥ 98%), CBT, CBD, CBG, CBGA, delta-8-Tetrahydrocannabinol (Δ^8^ THC), CBN, CBC, and CBDV were purchased from Cayman Chemical Company (Ann Arbor, MI, United States). Recombinant human kynurenine-3-monooxygenase (KMO) was purchased from Cosmo Bio USA (Carlsbad, CA, USA). The Series S Sensor Chip CM5 was purchased from GE Healthcare (Chicago, IL, USA). Amine coupling kit and buffers (HBS-EP+ and HBS-P+) were purchased from Cytiva (Marlborough, MA, USA). Phosphate buffered saline (PBS) and dimethyl sulfoxide (DMSO) were purchased from Thermo Fisher Scientific (Waltham, MA, USA).

### 2.2. Preparation of Cannflavin A (CFA)

CFA was purified from mature hemp aerial parts (*C. sativa* L.), including the leaves and flowers harvested at a hemp farm (Zhanyi District, Kunming, Yunnan, China) between July and September. The tetrahydrocannabinol level in the hemp material was less than 0.3% and a voucher specimen (220203) is deposited at Yunnan Hemp Mon Pharmaceutical Co., Ltd.; Kunming, Yinan, China. The hemp aerial parts were pulverized and passed through a sieve (#40–80 mesh). Hemp fine powder (300 kg) was extracted twice with 80% aqueous cold ethanol (8:1; *v*/*v*) at a stirring speed of 50 rpm for 2 h. The ethanol filtrate was combined, and the ethanol content was reduced to 50% to obtain a crude ethanolic extract. This extract was further purified with a chromatographic method with a macroporous adsorbent resin HPD700 (Bon Absorber Technology Co.; Cangzhou, Hebei, China) eluting with 53% aqueous ethanol (5 column volumes). The resulting fractions were combined, and the solvent was removed in vacuo to afford a crude extract of CFA (4.45 kg). The CFA crude ethanolic extract was then placed in a reaction vessel (20 L) and dissolved with hexane (4.5 L) at 40 °C, followed by filtration with primary and secondary filters to remove insoluble substances. After three times of filtration, solvents of the filtered solution were removed to obtain a yellow solid (52.28 g). Next, the yellow solid was dissolved in a mixture of ethyl acetate and acetone (5:1; *v*/*v*; 10 mL) and placed at 4 °C for 3 h to allow crystallization. Then, the crystalized solid was filtered and washed with cold ethyl acetate (20 mL) for three times to afford the purified CFA fraction. This CFA fraction was dried in an oven under reduced pressure for 36 h to obtain purified CFA (38.73 g).

### 2.3. HPLC Analysis of CFA

The purity of CFA was determined by an HPLC method. The purified CFA sample was measured (10.0 mg) in a conical flask and dissolved in 95% aqueous ethanol (10 mL) by ultrasonication for 5 min, followed by transferring to a volumetric flask (100 mL). The CFA standard (purity > 98%) was prepared at a concentration of 100 μg/mL. The HPLC analysis was performed on a Shimazu instrument with a Sepax GP-C18 column (4.6 × 150 mm, 3 μM) with a column temperature of 28 °C. The detection wavelength was 220 nm, and the injection volume was 10 μL. An isocratic solvents system (70% of aqueous acetonitrile with 0.1% formic acid for 20 min) was used for the elution.

### 2.4. Enzyme Activity Assay

The inhibitory effects of compounds from *C. sativa*. (CFA, CBT, CBD, CBG, CBGA, Δ^8^ THC, CBN, CBC, and CBDV) on the KMO enzyme were determined. The enzymatic assay was performed in duplicate. Ro 61-8048 was used as the positive control. The test compounds were dissolved in DMSO at 100 mM and diluted to 1 mM in assay buffer. A reaction mixture of assay buffer (50 μL), human KMO (20 μg/mL; 50 μL), test compounds (1 mM; 10 μL), and substrate (40 μL NADPH (10 mM) plus L-Kyn (20 mM)) were incubated in a 96-well plate at room temperature for 1.5 h. The absorbance was recorded at 340 nm. The enzyme activity was expressed as % activity as follows:

$$\%\;\mathrm{Activity}=[({\mathrm{Abs}}_{\mathrm{sample}}-{\mathrm{Abs}}_{\mathrm{blank}})/\left({\mathrm{Abs}}_{\mathrm{control}}-{\mathrm{Abs}}_{\mathrm{blank}}\right)]\times 100$$

% Inhibition was calculated by subtracting the % activity from 100%. The ${\mathrm{IC}}_{50}\;$ value of CFA and the known inhibitor, Ro 61-8048, were obtained through graphical analysis using GraphPad Prism.

### 2.5. Molecular Docking

Molecular docking was conducted to explore the binding mode between the KMO inhibitors and protein. The crystal structure of human KMO (resolution: 2.1Å; PDB ID: 5X68) was obtained from the RCSB Protein Data Bank (https://www.rcsb.org; accessed on 20 May 2022). The 3D chemical structures of CFA and Ro 61-8048 were obtained from the PubChem Database (https://pubchem.ncbi.nlm.nih.gov; accessed on 20 May 2022) and converted into the pdb format by the software UCSF Chimera. AutoDock Tools 1.5.6 software was used to remove water molecules, add hydrogens and charges, and to modify ligand torsions. In addition, the structure of the KMO protein was prepared by removing water molecules, adding hydrogens and charges, and assigning AD4-type atoms. A grid box was generated based on a previously published docking site of Ro 61-8048 [21]. The ligands were independently docked to the KMO protein using the long genetic algorithm parameter (maximum number of evaluations = 25 million). Binding parameters, including estimated free binding energy (kcal/mol), estimated inhibition constant; Ki (μM), final intermolecular energy (kcal/mol), electrostatic energy (kcal/mol), unbound system’s energy (kcal/mol), and total internal energy (kcal/mol) were predicted. The ligand-protein with the lowest binding energy was selected for further visualization and analysis by using Discovery Studio Version 4.5.

### 2.6. SPR Immobilization

Recombinant human KMO protein was diluted to 20 μg/mL in sodium acetate buffer (10 mM; pH 8). The protein was manually immobilized on a Series S Sensor Chip CM5 based on an amine coupling method with minor modification.

### 2.7. SPR Assay

An SPR assay was conducted on the Biacore T200 SPR instrument (Cytiva; Marlborough, MA, USA). HBS-P+ (20 mM; 0.02 M HEPES, 0.3 M NaCl, 0.1% *v*/*v* surfactant P20; pH 7.4) containing 3% DMSO was used as the running buffer. CFA stock solution (10 mM; dissolved in DMSO) was diluted into a series of concentrations ranging from 9.5 to 300 μM in the running buffer. Ro 61-8048 stock solution (6 mM; dissolved in DMSO) was diluted into a series of concentrations ranging from 1.25 to 20 μM in the running buffer. One sample cycle consisted of 60 s of contact time (association phase) and 60 s of dissociation time (dissociation phase) with a flow rate of 100 μL/min. To compensate for the bulk effects of DMSO, solvent correction cycles consisting of eight correction points (2.5–3.8% of DMSO) were conducted before and after every eight sample cycles. BIA Evaluation Software Version 4.1 (GE Healthcare, Chicago, IL, USA) were used to obtain parameters, including association rate $\left(K_{on}\right)$, disassociation rate $\left(K_{off}\right)$, and the equilibrium dissociation constants $\left(K_{D}\right)$.

### 2.8. Competitive SPR Assay

A competitive SPR binding assay was conducted to explore the binding site of CFA as compared to the known KMO inhibitor (Ro 61-8048) using our previously established methods with minor modifications [22]. HBS-P+ (20 mM; 0.02 M HEPES, 0.3 M NaCl, 0.1% *v*/*v* surfactant P20; pH 7.4) containing 3% DMSO was used as the running buffer. CFA was prepared as a final concentration of 75 μM in running buffer. Ro 61-8048 was prepared as a final concentration of 5 μM in running buffer. A mixture of solution containing CFA (75 μM) and Ro 61-8048 (5 μM) was prepared. The experimental RU values from CFA and Ro 61-8048 were collected to calculate the fractional occupancy (FO) of both compounds.

$$\mathrm{FO}\left(A\right)=\frac{1}{1+\frac{{\mathrm{KD}}_{A}}{C_{A}}\left(1+\frac{C_{B}}{{\mathrm{KD}}_{B}}\right)};\mathrm{FO}\left(B\right)=\frac{1}{1+\frac{{\mathrm{KD}}_{B}}{C_{B}}\left(1+\frac{C_{A}}{{\mathrm{KD}}_{A}}\right)}$$

where $C_{A}$ and $C_{B}$ are the respective concentrations of both binders, and ${\mathrm{KD}}_{A}$ and ${\mathrm{KD}}_{B}$ represent the equilibrium dissociation constants of the binders. The FO values were used to calculate the theoretical RU representative of competing compounds using the following equation [23]:

$$R_{\mathrm{observed}}=\mathrm{FO}\left(A\right)\cdot R_{\max}\left(A\right)+\mathrm{FO}\left(B\right)\cdot R_{\max}\left(B\right)$$

## 3. Results

### 3.1. Preparation of CFA at a Gram-Scale

CFA was purified at a gram-scale (38.73 g) from the hemp aerial parts (300 kg of raw material) via chromatographic methods with a yield of 0.013% (Table 1). The chemical structure of CFA was confirmed by nuclear magnetic resonance (NMR) spectroscopic data: ^1^H NMR (600 MHz, DMSO) δ 13.21 (s, 1H), 10.76 (s, 1H), 9.93 (s, 1H), 7.52 (s, 2H), 6.93 (d, J = 8.2 Hz, 1H), 6.86 (d, J = 2.4 Hz, 1H), 6.54 (s, 1H), 5.17 (t, J = 6.8 Hz, 1H), 5.01 (s, 1H), 3.89 (s, 3H), 3.22 (d, J = 6.7 Hz, 2H), 1.98 (d, J = 5.9 Hz, 2H), 1.91 (s, 2H), 1.72 (s, 3H), 1.56 (s, 3H), and 1.50 (s, 3H).

^13^C NMR (151 MHz, DMSO) δ 181.92, 163.49, 161.90, 158.49, 155.18, 150.74, 148.12, 134.24, 130.72, 124.19, 122.14, 121.75, 120.36, 115.87, 111.05, 110.21, 103.63, 103.25, 93.34, 56.04, 39.36, 26.28, 25.53, 21.07, 17.59, and 16.01 (See the NMR spectra in the Supplementary Materials Figures S1 and S2).

**Figure 2:**
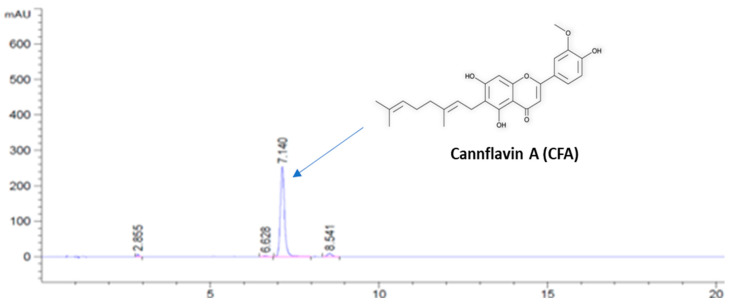
The HPLC chromatographic profile and chemical structure of purified CFA. Chromatography column condition: Sepax GP-C18 column (4.6 × 150 mm, 3 μm) with a column temperature of 28 ℃. Elution method: isocratic solvents system with 70% of aqueous acetonitrile with 0.1% formic acid for 20 min.

As shown in Figure 2, the purity of CFA (at the retention of 7.1 min) was determined as 96.45% by the HPLC analysis. This purification method was developed to afford CFA from hemp extracts at a gram-scale, which provide sufficient quantity for the biological evaluations of this unique flavonoid.

### 3.2. CFA Inhibits KMO Enzyme Activity

**Figure 3:**
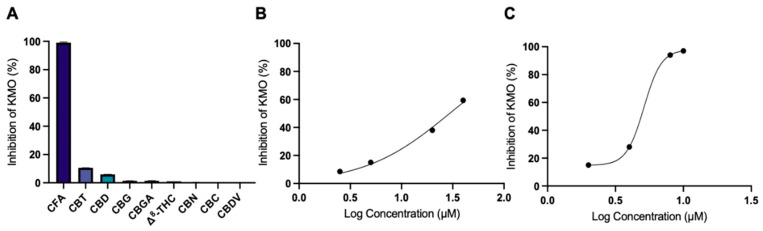
Inhibitory effects of *C. sativa* compounds on KMO. (**A**) Percent inhibition of *C. sativa* compounds on KMO enzyme activity at 100 μM. (**B**) Percent inhibition of KMO by CFA at 2.5–40 μM. (**C**) Percent inhibition of KMO by Ro 61-8048 at 2–10 μM.

A panel of compounds from *C. sativa* were evaluated for their in vitro inhibition on human KMO. CFA, CBT, CBD, CBG, CBGA, Δ^8^ THC, CBN, CBC, and CBDV at 100 μM exhibited 99, 11, 6, 1, 1, 1, 0, 0, and 0% inhibition, respectively (Figure 3A). CFA exhibited the highest inhibition percentage (99%) of the KMO protein at 100 μM, prompting further evaluation of the IC_50_ value. CFA displayed an IC_50_ value of 29.4 μM with 59, 38, 15, and 8.5% inhibition at 40, 20, 5, and 2.5 μM, respectively (Figure 3B; Table 2). CFA is comparable to Ro 61-8048, a known KMO inhibitor which displayed an IC_50_ value of 5.1 μM (Figure 3C; Table 2).

### 3.3. Molecular Docking Analysis

**Figure 4:**
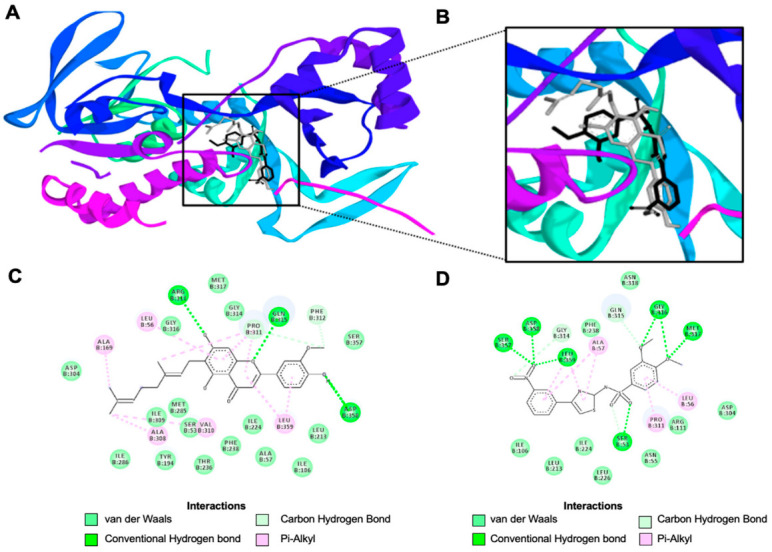
Molecular docking interactions of CFA and Ro 61-8048 with KMO. (**A**) Visualization of the docking site between CFA and Ro 61-8048 on the KMO protein. (**B**) Enlarged depiction of the ligand docking site on KMO with CFA colored in gray and Ro 61-8048 colored in black. (**C**) Predicted two-dimensional binding interactions between CFA and KMO. (**D**) Predicted two-dimensional binding interactions between Ro 61-8048 and KMO.

A docking analysis was performed between CFA and the KMO using AutoDock Tools (1.5.6). CFA was docked to the protein using a previously reported binding site for Ro 61-8048 [16] (Figure 4A,B). Ro 61-8048 was shown to bind to the same binding pocket on KMO as demonstrated by the theoretical location where the ligand bound to the enzyme (Figure 4A,B). Ligand-protein interaction analysis revealed that CFA formed conventional hydrogen bonds with ARG111, GLN315, and ASP358, carbon hydrogen bonds with PRO311 and PHE312, and pi-alkyl bonds with LEU 56, ALA169, ALA308, VAL310, and LEU 359 (Figure 4C). Ro 61-8048 formed molecular interactions with the KMO enzyme through conventional hydrogen bonds with SER53, GLY316, MET317, SER 357, ASP 358, and LEU 358, carbon hydrogen bond with GLY 314 and GLN 315, and pi-alkyl bonds with LEU 56, ALA 57, and PRO 311 (Figure 4D).

The binding parameters of CFA and Ro 61-8048 were evaluated using AutoDock Tools revealing ligand-protein interactions. CFA bound to KMO with a binding energy of −7.95 kcal/mol, an inhibition constant of 1.49 μM, intermolecular energy of −10.93 kcal/mol, electrostatic energy of −0.38 kcal/mol, unbound system’s energy of −1.82 kcal/mol, and total internal energy of −1.82 kcal/mol (Table 3). The binding parameters of Ro 61-8048 demonstrated a strong binding energy of −9.75 kcal/mol, an inhibition constant of 0.07 μM, intermolecular energy of −11.84 kcal/mol, electrostatic energy of −1.34 kcal/mol, unbound system’s energy of −0.63 kcal/mol, and total internal energy of −0.63 kcal/mol (Table 3).

### 3.4. An SPR Method Was Validated for Measurement of Binding Affinity

**Figure 5:**
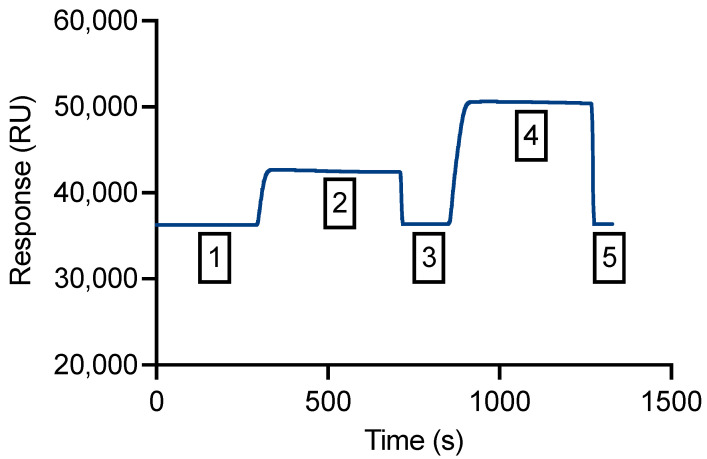
The validation of the SPR assay to measure the binding affinity of CFA and Ro 61-8048 to the recombinant KMO protein. KMO protein was immobilized on the SPR chip and HBS-EP+ (10 mM; 0.02 M HEPES, 0.3 M NaCl, 0.006 M EDTA, 0.1% *v*/*v* surfactant P20; pH 7.4) was used as the running buffer. Around 3200 RUs of the recombinant KMO protein were immobilized on the flow cell 2. Blank immobilization was performed on the flow cell 1. Immobilization phases 1–5 are represented as numbered boxes.

A label-free biosensor-based binding method using the SPR technique was optimized to measure the binding affinity of CFA and the KMO protein. As shown in Figure 5, a stable baseline (phase 1) and a responsive effect in the RU was observed (phase 2). After a wash with ethanolamine hydrochloride (1 M; pH 8.5) was performed (phase 3), response was observed as recombinant KMO protein, 20 μg/mL) was injected and coupled to the surface matrix with a contact time of 2100 s and flow rate of 5 μL/min (phase 4). A full binding association and dissociation was observed as ethanolamine hydrochloride (1 M; pH 8.5) was used to deactivate unreacted NHS-esters and remove any remaining electrostatically bound ligand with a contact time of 420 s and a flow rate of 10 μL/min (phase 5). This suggested that the recombinant KMO protein was successfully coated on the SPR sensor chip for the further measurement of analytes’ binding affinity.

**Figure 6:**
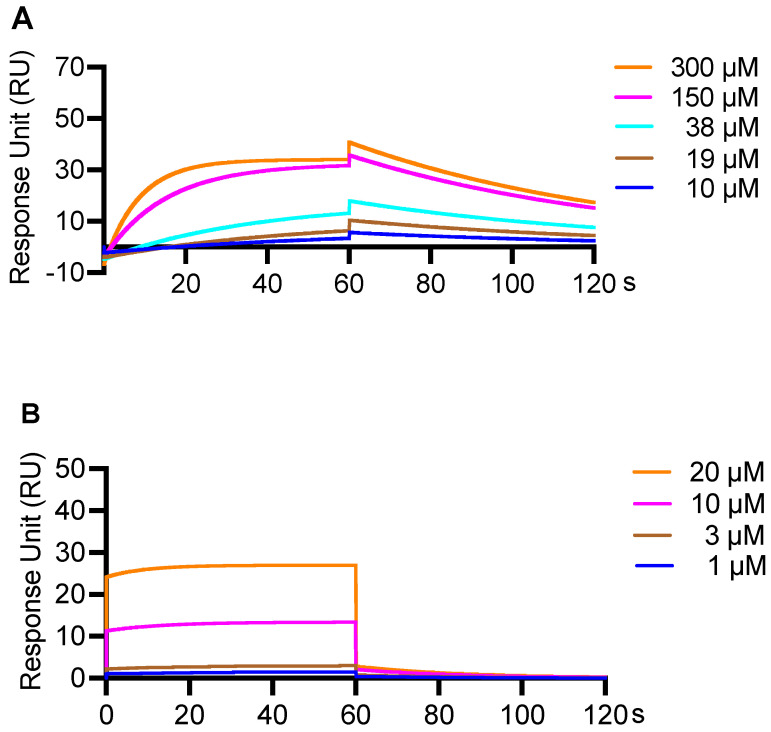
SPR binding of CFA and Ro 61-8048 to KMO. The sensorgrams display the binding reaction over time for (**A**) CFA (10, 19, 38, 150, and 300 μM) and (**B**) Ro 61-8048 (1, 3, 10, and 20 μM).

Next, the independent binding profiles of CFA and Ro 61-8048 to KMO were studied by the SPR assay, which resulted in a series of concentration response curves. CFA’s binding response unit (RU) exhibited a concentration dependent increase at 10, 19, 38, 150 and 300 μM, with RU values of 5.6, 10.4, 17.9, 35.7, and 40.7, respectively (Figure 6A). Ro 61-8048’s binding response unit (RU) similarly showed concentration dependence where the RU increased at 1, 3, 10, and 20 μM to 1.48, 2.99, 13.4, and 27.0, respectively (Figure 6B). Further, the binding profile including association rate $\left(K_{on}\right)$, dissociation rate $\left(K_{off}\right)$, and dissociation constant $\left(K_{D}\right)$, were obtained (Table 4). The association and dissociation rates of CFA were $0.3\times 10^{3}\;1/\mathrm{Ms}$ and $14.2\times 10^{-3}\;1/s$. CFA showed comparable binding capacity with Ro 61-8048 with dissociation constants of $4.1\times 10^{-5}\;M$ vs. $1.1\times 10^{-5}\;M$.

### 3.5. Competitive SPR Assay

**Figure 7:**
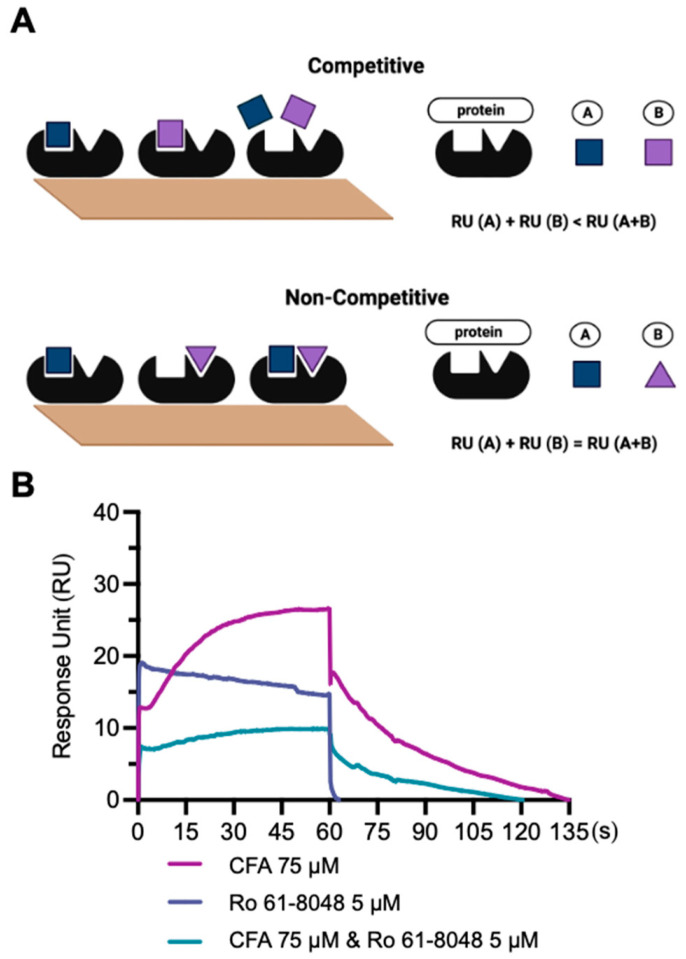
Competitive SPR binding assay between CFA and Ro 61-8048 on the KMO protein. (**A**) Two binding modes: RU (A) + RU (B) < RU (A + B) indicates competitive binding and RU (A) + RU (B) = RU (A + B) for non-competitive binding. (**B**) SPR sensorgram for CFA (75 μM), Ro 61-8048 (5 μM), and CFA (75 μM) combined with Ro 61-8048 (5 μM) binding with KMO.

The binding site on KMO was further explored by using a competitive SPR assay. Single binders, including CFA and Ro 61-8048, as well as the combination of the two ligands were applied (Figure 7). As shown in Figure 7A, we can distinguish the theoretical binding mode (either competitive or non-competitive) by comparing the theoretical RU values. The experimental RU values for CFA (75 μM) and Ro 61-8048 (5 μM) were 27 and 19 RU, respectively (Figure 7B; Table 4). When CFA (75 μM) and Ro 61-8048 (5 μM) were combined, the RU value was 7 (Figure 7B; Table 5), which is closer to the theoretical competitive binding RU value (11), indicating that CFA may have the same binding site on KMO as compared to Ro 61-8048. Consistently, the molecular docking data also supported that CFA bound to a similar site on KMO as Ro 61-8048. To date, this is the first study exploring the mechanism of inhibition on KMO by a *C. sativa* compound using the SPR assays, which provides useful information for the further development of KMO inhibitors.

## 4. Discussion

KP dysregulation, including an increase in KMO activity, is associated with numerous pathological presentations, including neurological disorders, autoimmune diseases, inflammation, cancer, and stroke [1]. KMO acts as a regulator of the KP, with imbalances leading to peripheral inflammation and diseases of the central nervous system [24]. Specifically, KMO inhibition has been shown to shift the KP from neurotoxic products to neuroprotective which functions in regulating KP metabolism [24,25]. The identification of compounds possessing inhibitory action against KP enzymes is of great research interest for the development of pharmaceuticals against disease conditions aiming to normalize the KP [26].

Naturally derived phytochemicals, such as flavonoids have previously shown beneficial properties by targeting enzymes involved in KP metabolism. The flavonoid NSC 36398 (dihydroquercetin, taxifolin) was shown to be a selective inhibitor of tryptophan 2,3-dioxygenase (TDO), the first enzyme in the KP, with implications for cancer immunosuppression [27]. In addition, the citrus flavonoid hesperidin, lowered kynurenine levels in the hippocampus and prefrontal cortex in a subchronic and mild social defeat mouse model [28]. Further, phytocannabinoids, including CBD and THC were identified as suppressors of IDO activity in human peripheral mononuclear cells [29]. Moreover, CBD and CBG were recently found to elicit neuroprotective effects in a rat hypothalamic cell line of oxidative stress induced with hydrogen peroxide, via a reduction of the 3-hydroxykynurenine/kynurenic acid ratio [30]. To date, our current study is the first to evaluate the direct anti-KMO effects of phytocannabinoids including the major one (CBD) and a collection of minor ones, as well as a non-phytocannabinoid compound, CFA.

The *C. sativa* derived flavonoid, CFA, has been reported to confer numerous biological activities such as antioxidant, anti-inflammatory, and neuroprotective properties [20]. Further, CFA was recently found to induce cytotoxicity and exhibit synergistic effects in a human bladder cell line [31]. Yet, CFA’s biological actions are currently limited in literature, and CFA has not previously been evaluated in regard to the KP metabolism. Research on CFA’s biological activities might be hindered given that the level of CFA in *C. sativa* is low [19]. Therefore, we developed a method to extract and purify CFA at a gram-scale, and CFA served as our compound of interest in assessing KMO inhibition through in vitro assays, *in-silico* approach, and biophysical techniques. Our findings are the first to demonstrate moderate inhibition of KMO by CFA, as well as slight inhibitory effects of phytocannabinoids. CFA was identified to be the most potent inhibitor of KMO enzymatic activity, although at the micromolar level, compared to a panel of phytocannabinoids. The greater inhibitory effect of CFA on KMO than the phytocannabinoids may be due to its chemical structure, including a C10 geranyl group attached to a flavone A-ring (Figure 2), which has been hypothesized to contribute to CFA’s biological activities [32]. Further, CFA demonstrated a binding affinity to KMO comparable to Ro 61-8048 in an SPR binding analysis, which validated initial molecular docking evaluations. Lastly, a competitive SPR analysis revealed competitive, rather than non-competitive binding of CFA and Ro 61-8048 to the KMO protein, which provides insight into the binding mode of CFA.

CFA’s effect on KMO was comparable to the well-studied inhibitor, Ro 61-8048, indicating promise for future drug development as a lead compound against KMO-related disease conditions. Ro 61-8048 has been shown to elicit an increased production of the KP metabolite, KYNA, in animal models of cerebral ischemia and autoimmune encephalomyelitis [33,34]. Further, Ro 61-8048 reduced dyskinesia when administered with L-DOPA/benserazide in 1-methyl 4-phenyl-1,2,3,6-tetrahydropyridine monkeys [35]. Additionally, Ro 61-8048 was shown to lower pro-inflammatory cytokines and nitrites via KMO inhibition in BV2 microglia cells treated with lipopolysaccharides [36]. As our findings demonstrate similarities with Ro 61-8048, employing cell and animal models may further confirm whether CFA’s effect on KMO has similar pharmacological implications.

While CFA has previously been shown to have beneficial therapeutic properties, it remains poorly studied in the context of tryptophan metabolism. Thus, CFA may be a compound of interest in relation to the identification of lead compounds against neuroinflammation and neurodegeneration. As CFA demonstrated inhibitory effects on KMO, the potential effect of other *C. sativa* flavonoids, such as cannflavins B and C, should also be studied. In addition, it has been reported that some KMO inhibitors independently increase cytoplasmic hydrogen peroxide production, specifically, inhibitors comparable to the native substrate L-Kyn [1]. As CFA is structurally dissimilar to L-Kyn, we hypothesize that it would not induce additional hydrogen peroxide production. However, whether CFA and other *C. sativa* metabolites increase hydrogen peroxide alone warrants further evaluation to better elucidate the capacity for these compounds to be of clinical significance. Additional cell and in vivo studies are warranted to further evaluate whether *C. sativa* derived phytochemicals show promise as mediators targeting the KP.

## 5. Conclusions

In summary, a chromatographic method was developed to enable obtaining purified CFA from hemp aerial material at a gram-scale, which facilitated the biological evaluation of CFA. Our findings utilized a combination of enzymatic assays, *in-silico* modeling, and biophysical techniques to characterize the biological effects of *C. sativa* compounds on KMO. Our study is the first to determine the inhibitory activity of *C. sativa*-derived compounds, including major and minor phytocannabinoids, as well as the flavonoid, CFA, on KMO. In addition, we used a biophysical tool (i.e., SPR) to elucidate the mechanism of CFA’s binding profile to the KMO protein. CFA and a panel of phytocannabinoids were evaluated for the KMO inhibitory activity, and CFA showed the most potent activity. CFA’s mechanism of action was further studied by its binding mode (by molecular docking) and affinity (by SPR assays), which revealed that CFA and Ro 61-8048 shared a similar binding behavior. This is the first study using an SPR immobilization method to enable studying the KMO binding profile of active ligand from *C. sativa*. Nevertheless, some limitations, such as the fact that the anti-KMO effects of *C. sativa* phytochemicals were not assessed in an in vivo or cell-based model, need to be addressed. In addition, it is unknown whether the KMO inhibitors from *C. sativa* can exert their effects in the brain by passing the blood brain barrier. Therefore, further studies are warranted as it is unknown whether CFA exerts its inhibition of KMO peripherally or in the central nervous system by penetrating the blood brain barrier. In addition, cell culture and animal-based models are warranted to elucidate whether CFA’s inhibition of KMO can shift the kynurenine pathway toward the neuroprotection branch, which is critical for the further development of *C. sativa*-derived KMO inhibitors as therapeutics for neurodegenerative or inflammatory diseases. The evaluations of the biological effects of CFA on KMO support the promise for *C. sativa* compounds, such as CFA, to be utilized as lead compounds in the development of KMO inhibitors.

## Figures and Tables

**Table 1:** Preparation of CFA from hemp aerial material at a gram-scale.

Raw Hemp Mass	Dry Hemp Mass	CFA		
Kg	Kg	g	Purity ^a^	Yield
300	262.32	38.73	96.45%	0.013%

^a^ The purity of CFA was determined by the HPLC analysis.

**Table 2:** The inhibitory effects IC_50_ values of CFA and Ro 61-8048 against the KMO enzyme.

Inhibitor	IC_50_ (μM)
CFA	29.4
Ro 61-8048	5.1

**Table 3:** Molecular docking binding parameters of CFA and Ro 61-8084 with the KMO protein.

Binding Parameters	CFA	Ro 61-8048
Estimated Free Binding Energy (kcal/mol)	−7.95	−9.75
Estimated Inhibition Constant; Ki (μM)	1.49	0.07
Final Intermolecular Energy (kcal/mol)	−10.93	−11.84
Electrostatic Energy (kcal/mol)	−0.38	−1.34
Unbound System’s Energy (kcal/mol)	−1.82	−0.63
Total Internal Energy (kcal/mol)	−1.82	−0.63

**Table 4:** Association $\left(K_{on}\right)$ rate, dissociation $\left(K_{off}\right)$ rate, and dissociation constant $\left(K_{D}\right)$ for CFA and Ro 61-8048.

Ligand	$K_{on}\;\left(\times 10^{3},\;1/Ms\right)\;$	$K_{off}\;\left(\times 10^{-3},\;1/s\right)$	$K_{D}\;\left(\times 10^{-5},\;M\right)$
CFA	0.3	14.2	4.1
Ro 61-8048	3.7	38.5	1.1

**Table 5:** Competitive SPR binding assay experimental and theoretically calculated binding RU for CFA, Ro 61-8048, and CFA and Ro 61-8048 combined.

Ligand	Experimental RU	Theoretically Calculated RU	
		**Non-Competitive**	**Competitive**
CFA	27		
Ro 61-8048	19		
CFA + Ro 61-8048	7	46	11

## Data Availability

Raw data obtained in this study are available from the corresponding authors upon reasonable request.

## Supplementary Materials

The following supporting information can be downloaded at: https://www.mdpi.com/article/10.3390/biology11101416/s1. 1H- and 12C-NMR spectra of CFA: Figures S1 and S2.
