# Supplementary material for: Gram-Scale Preparation of Cannflavin A from Hemp (Cannabis sativa L.) and Its Inhibitory Effect on Tryptophan Catabolism Enzyme Kynurenine-3-Monooxygenase
Source: Biology (Basel). 2022 Sep 28;11(10):1416. doi: 10.3390/biology11101416 (PMC9598531; doi:10.3390/biology11101416)

Supplementary Materials

Gram-Scale Preparation of Cannflavin A From Hemp (*Cannabis sativa* L.) and Its Inhibitory Effect on Tryptophan Catabolism Enzyme Kynurenine-3-Monooxygenase

Figure S1. ^{1}H-NMR spectrum of CFA.

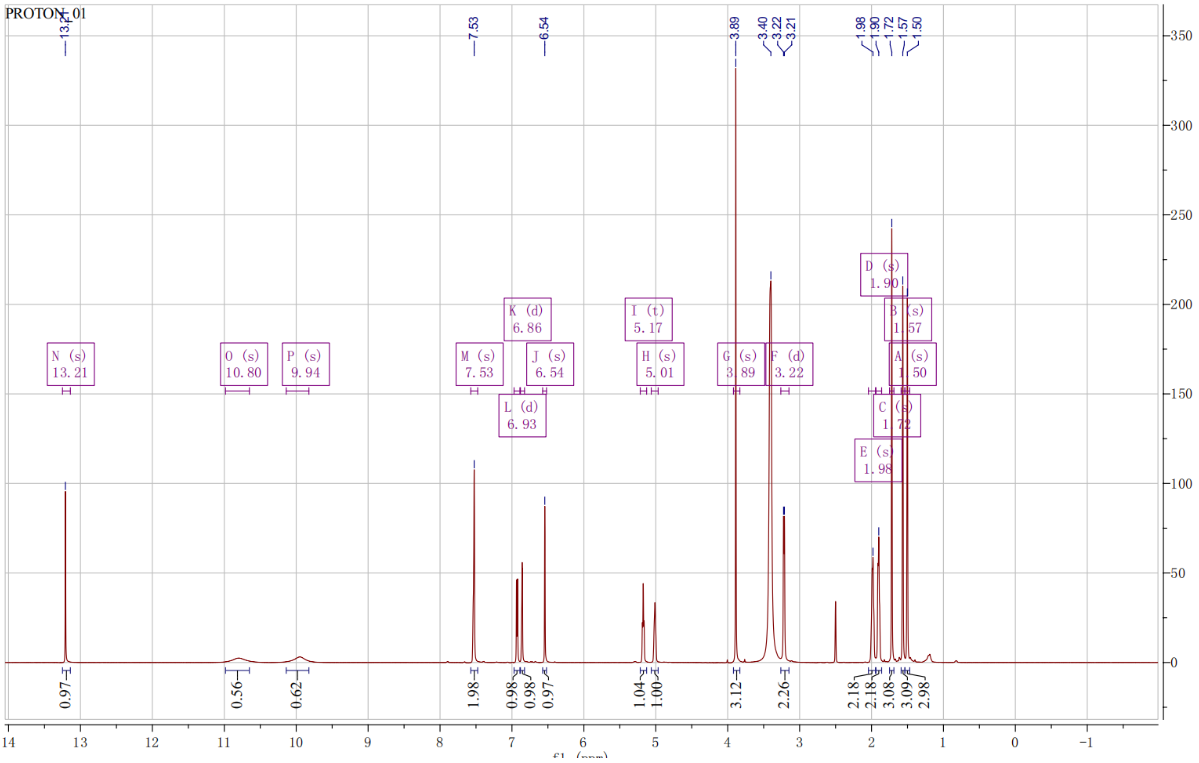

Figure S2. ^{12}C-NMR spectrum of CFA.

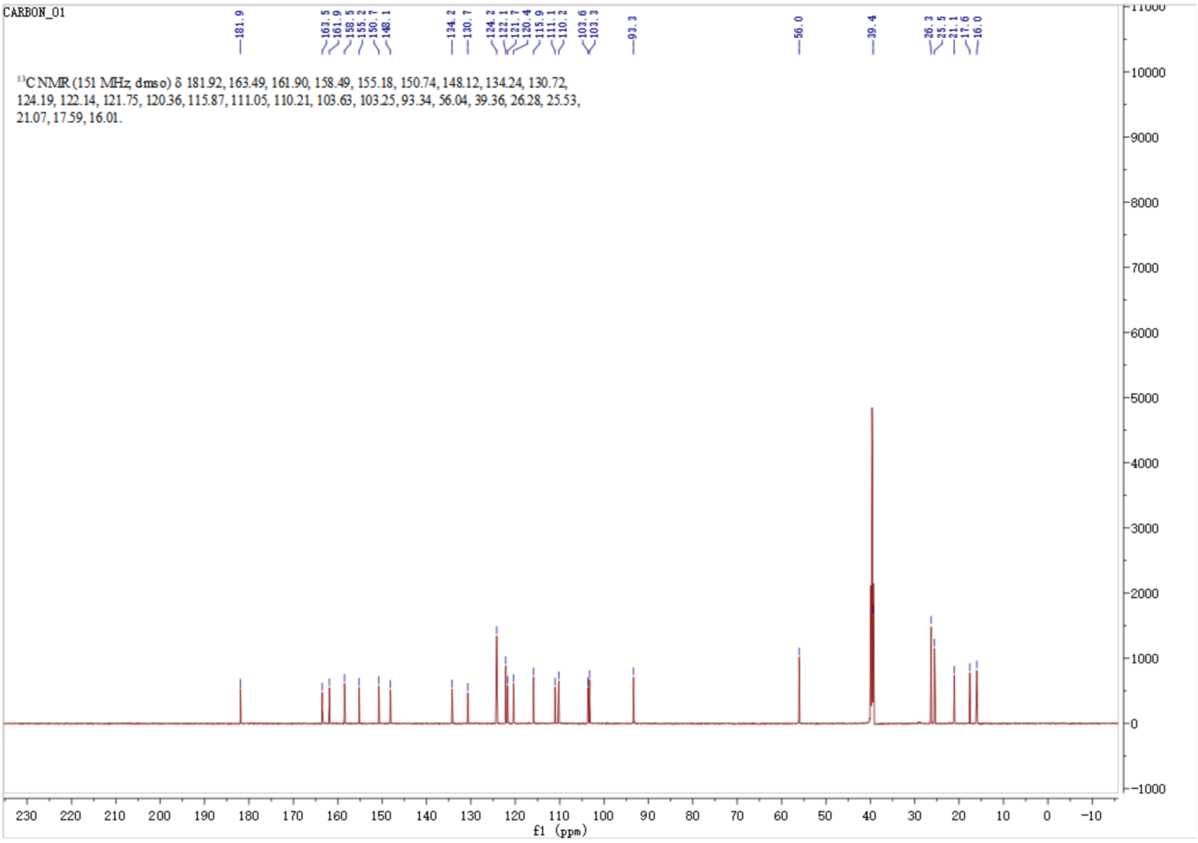

Supplement: Supplementary file 1 [file biology-11-01416-s001.zip › biology-1896269-supplementary.pdf]
